# Analysis of 33,616 urinary stone cases: novel findings on renal transplantation impact, comorbidity profiles, and composition patterns

**DOI:** 10.3389/fimmu.2026.1757548

**Published:** 2026-03-19

**Authors:** Lian Peng, Xingyu Zhou, Jialin Liu, Jierong Chen, Jiale Zhang, Feiyang Tan, Baolin Li, Ying Liang, Qianjun Li, Zhenglin Chang, Lin Yu, Ming Zhao

**Affiliations:** 1 Department of Urology, Zhujiang Hospital of Southern Medical University, Guangzhou, Guangdong, China; 2 Department of Urology, Yuebei People’s Hospital Affiliated to Shantou University Medical College, Shaoguan, China; 3 Department of Urology and Guangdong Key Laboratory of Urology, The First Affiliated Hospital of Guangzhou Medical University, Guangzhou, China; 4 Department of Clinical Laboratory, National Center for Respiratory Medicine, National Clinical Research Center for Respiratory Disease, State Key Laboratory of Respiratory Disease, Guangzhou Institute of Respiratory Health, The First Affiliated Hospital of Guangzhou Medical University, Guangzhou, Guangdong, China

**Keywords:** calcium oxalate, carbonate apatite, propensity score matching, renal transplantation, stone disease, urinary stone composition

## Abstract

**Background:**

Urinary stone composition in renal transplant recipients remains poorly characterized, despite their distinct metabolic and urological profiles. Understanding stone composition patterns in this population is crucial for targeted management and recurrence prevention strategies.

**Methods:**

This retrospective observational study analyzed 33,616 urinary stone samples from two hospitals in Southern China (April 2014-December 2024). We compared stone composition between 69 renal transplant recipients with nephrolithiasis and 33,547 non-transplant stone-forming controls using Fourier Transform-Infrared Spectrometry. Propensity score matching (1:2) was performed to balance baseline characteristics. Logistic regression with progressive adjustment and comprehensive subgroup analyses were conducted to assess associations between transplantation and stone composition.

**Results:**

After propensity score matching, transplant recipients with nephrolithiasis demonstrated markedly different stone composition patterns compared to controls. Before matching, carbonate apatite stones were substantially more prevalent among stone formers in the transplant group (37.7% vs. 16.4%, p<0.001), while calcium oxalate monohydrate stones were less common (39.1% vs. 63.7%). After matching with 139 controls, these differences persisted and intensified (carbonate apatite: 37.7% vs. 15.8%; calcium oxalate monohydrate: 39.1% vs. 66.9%, p=0.001). Renal transplantation with nephrolithiasis was associated with significantly lower proportion of calcium oxalate stones compared to non-transplant stone formers (OR 0.32, 95% CI: 0.17-0.57, p<0.001) and significantly higher odds of carbonate apatite stones (OR 3.22, 95% CI: 1.66-6.32, p<0.001).These associations remained robust across sensitivity analyses with progressive adjustment for confounders (adjusted OR for carbonate apatite: 2.82, 95% CI: 1.37-5.82) and multiple subgroups stratified by age, sex, and comorbidities. Additional factors associated with carbonate apatite included renal atrophy (OR 4.37, 95% CI: 1.49-13.17, p=0.007) and urinary tract stricture (OR 2.50, 95% CI: 1.06-5.75, p=0.032). Transplant recipients with nephrolithiasis also exhibited higher proportions of mixed stone composition (58.0% vs. 39.6%, p=0.018) and urological complications.

**Conclusions:**

Renal transplantation fundamentally alters urinary stone pathogenesis in patients who develop stones, with distinct compositional shifts toward carbonate apatite and away from calcium oxalate stones. These findings suggest the need for transplant-specific stone prevention protocols.

## Introduction

Urinary stone disease represents a significant clinical challenge worldwide, affecting up to 14.8% of the global population with substantial healthcare burden and recurrence rates (1). Stone composition analysis provides crucial insights into underlying metabolic abnormalities and guides management strategies, with calcium oxalate being the most prevalent stone type in the general population (2, 3). Recent large-scale studies have revealed complex patterns in stone composition related to demographics, comorbidities, and seasonal variations (4). However, renal transplant recipients represent a unique population with fundamentally altered physiology. The metabolic milieu of these patients—characterized by chronic immunosuppressive therapy, perturbed calcium-phosphate homeostasis, vitamin D dysregulation, and frequent urological complications—suggests distinct stone pathogenesis (5, 6). Post-transplant metabolic bone disease, altered parathyroid function, and medications such as calcineurin inhibitors and corticosteroids further complicate the picture (7). Despite growing recognition of stone disease as a significant post-transplant complication affecting 1-4% of recipients, with contemporary registry data confirming a 3-year incidence of approximately 1.7% (5), large-scale studies systematically comparing stone composition patterns between transplant recipients and matched controls using rigorous propensity score matching methods remain scarce (8, 9).

Given the distinct physiological, pharmacological, and anatomical profiles of renal transplant recipients, we hypothesized that among patients who form urinary stones, the composition profile would differ substantially from that in non-transplant patients. The interplay between immunosuppressive medications, metabolic alterations including hypercalciuria and hyperuricosuria, structural changes such as ureteral strictures, and recurrent urinary tract infections may shift stone formation patterns in ways not yet fully elucidated (10). Previous small-scale studies have suggested increased infection stone prevalence in transplant recipients, but comprehensive analyses with adequate control groups and adjustment for confounding factors are lacking (11). Understanding these compositional differences is essential for developing targeted evaluation, treatment, and recurrence prevention strategies in this vulnerable population, where stone disease can threaten graft function and patient outcomes.

Therefore, we conducted this large-scale retrospective observational study analyzing 33,616 urinary stone samples collected over a decade (2014-2024) to comprehensively characterize stone composition patterns in renal transplant recipients who developed nephrolithiasis with those in matched non-transplant stone formers. Using propensity score matching to minimize selection bias and rigorous statistical methods including progressive adjustment models and comprehensive subgroup analyses, we aimed to: (1) identify specific stone composition profiles associated with renal transplantation among stone-forming patients; (2) quantify the magnitude of these associations while controlling for potential confounders; (3) explore demographic and clinical factors that may modify these relationships; and (4) elucidate potential mechanistic pathways linking transplantation to these distinct stone composition patterns, thereby providing evidence-based guidance for the clinical evaluation, treatment, and prevention of stones in transplant patients (4, 12).

## Methods

### Study design and sample collection

**Figure 1:**
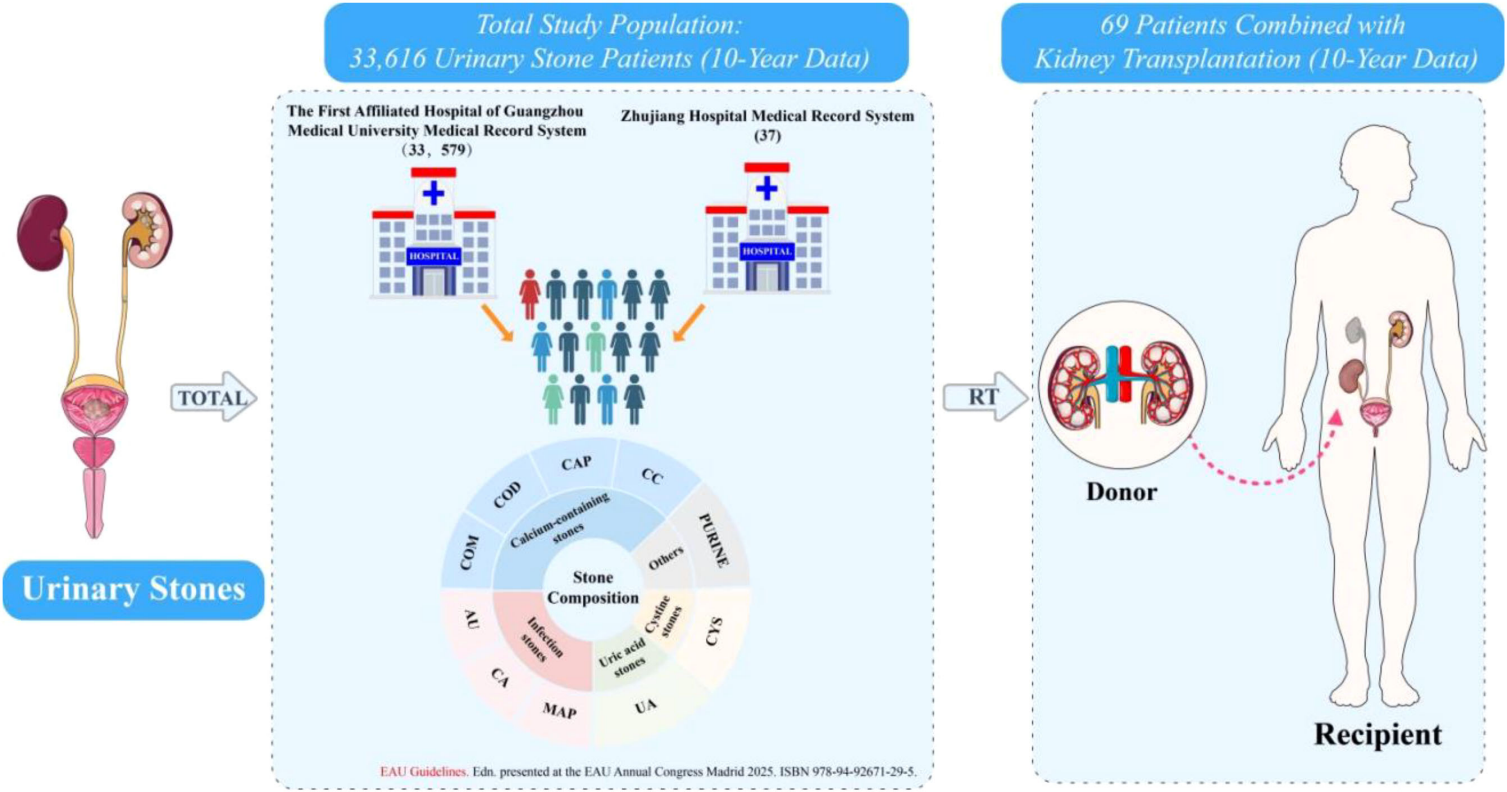
Study profile and baseline characteristics of kidney transplant recipients with urinary stones. The flowchart depicts the identification of kidney transplant recipients from a 10-year urinary stone disease database. Stone formers in this cohort were categorized into five groups based on their primary stone composition: calcium-containing stones, infectious stones, uric acid stones, cystine stones, and other stones.

This retrospective observational study analyzed urinary stone composition data from 33,616 samples collected at the First Affiliated Hospital of Guangzhou Medical University and Zhujiang Hospital of Southern Medical University between April 2014 and December 2024. The study population comprised exclusively patients who underwent surgical or spontaneous passage of urinary stones and subsequent compositional analysis. For each specimen, primary, secondary, and tertiary components were documented, along with patient demographics (age and sex) and detailed comorbidities. Patients were categorized into two groups: the renal transplantation group (patients with a history of kidney transplantation who subsequently developed urinary stones) and the control group (stone-forming patients without transplantation history) (**Figure 1**). Inclusion criteria were: (1) availability of complete stone composition analysis data, and (2) complete demographic and clinical information. Exclusion criteria included: (1) incomplete stone composition analysis, (2) missing critical baseline data, and (3) stones analyzed by methods other than infrared spectroscopy. Due to the retrospective nature of the study and the use of anonymized data, the requirement for informed consent was waived (ES-2025-K062). The data collection protocol adhered to the principles outlined in the Declaration of Helsinki.

### Stone composition analysis

In our laboratory, stone composition was examined using Fourier Transform-Infrared Spectrometry (Thermo). As per the European Association of Urology guidelines, stones were first categorized into seven primary types, including calcium oxalate (CaOx), calcium phosphate (CaP), uric acid (UA), magnesium ammonium phosphate (MAP, struvite), carbonate apatite (CA), ammonium urate (AU), and cystine (CYS) (13). Stones with MAP, CA, or AU were classified as infection stones. To conduct a more detailed analysis, we reorganized these elements into five primary categories: calcium-containing stones (including various forms of calcium oxalate monohydrate [COM], calcium oxalate dihydrate [COD], calcium phosphate [CaP], and calcium carbonate [CC]), infection stones (comprising magnesium ammonium phosphate [MAP], carbonate apatite [CA], and ammonium urate [AU]), uric acid stones (including uric acid [UA] and its derivatives), cystine stones (CYS), and other rare compositions. Every stone sample was examined for its primary, secondary, and tertiary components. For the primary statistical analysis comparing composition prevalence between groups, stones were classified based on their dominant (primary) component.

### Comorbidity assessment

The documentation and classification of comorbidities included urological conditions (urinary tract infection [UTI], hydronephrosis and effusion [UTHE], urinary tract stricture [UTS], renal insufficiency [RI], renal failure [RF], renal atrophy [RA], urinary tract malformation [UTM], hyperuricemia [HUA], gout, renal cyst [RC], renal transplantation [RT], tumor/neoplasm [T/N], neurogenic bladder [NB], and benign prostatic hyperplasia [BPH]), metabolic disorders (nephropathy [NP], fatty liver disease [FLD], hyperthyroidism [HT], hyperparathyroidism [HPT], hypothyroidism [HT-], diabetes mellitus [DM], hyperlipidemia [HLP]), cardiovascular diseases (hypertension [HTN], coronary heart disease [CHD], heart disease [HD], cerebrovascular disease [CVD]), respiratory conditions (chronic obstructive pulmonary disease [COPD]), gastrointestinal disorders (peptic ulcer disease [PUD]), autoimmune conditions (connective tissue disease [CTD]), hematological disorders (anemia [A]), and other systemic conditions (anxiety disorder [AD], sepsis). The presence or absence of each comorbidity was documented according to the patients’ medical records.

### Propensity score matching

To reduce confounding by indication and selection bias, we performed 1:2 propensity score matching using nearest-neighbor matching with a caliper width of 0.2 standard deviations. The propensity score was calculated using a logistic regression model that included age and sex as covariates. Balance diagnostics were assessed using standardized mean differences (SMD), with SMD <0.1 considered indicative of adequate balance. After matching, we verified that baseline characteristics were well-balanced between the transplant and control groups.

### Statistical analyses

All statistical analyses were performed using R and IBM SPSS Statistics (Version 25.0). Continuous variables were presented as mean ± standard deviation or median (interquartile range) depending on distribution normality, and compared using Student’s t-test or Mann-Whitney U test. Categorical variables were expressed as frequencies and percentages, and compared using chi-square test or Fisher’s exact test when appropriate. Univariate logistic regression analysis was performed to identify associations between renal transplantation and specific stone compositions, with results presented as odds ratios (OR) and 95% confidence intervals (CI). To assess the robustness of associations, we conducted sensitivity analyses using progressively adjusted models: Model 1 (unadjusted), Model 2 (adjusted for age and sex), and Model 3 (further adjusted for urinary tract stricture and renal atrophy for carbonate apatite stones). Subgroup analyses were performed stratified by age (<65 vs. ≥65 years), sex, comorbidities, and urological conditions, with interaction tests conducted using likelihood ratio tests. Forest plots were generated to visualize effect estimates across subgroups. All statistical tests were two-sided, and P<0.05 was considered statistically significant. Graphical visualization was performed using the ‘ggplot2’ package in R, with subsequent color and layout refinements made in Adobe Illustrator, as previously described (14–16).

## Results

### Baseline characteristics and stone composition patterns before and after propensity score matching

**Figure 2:**
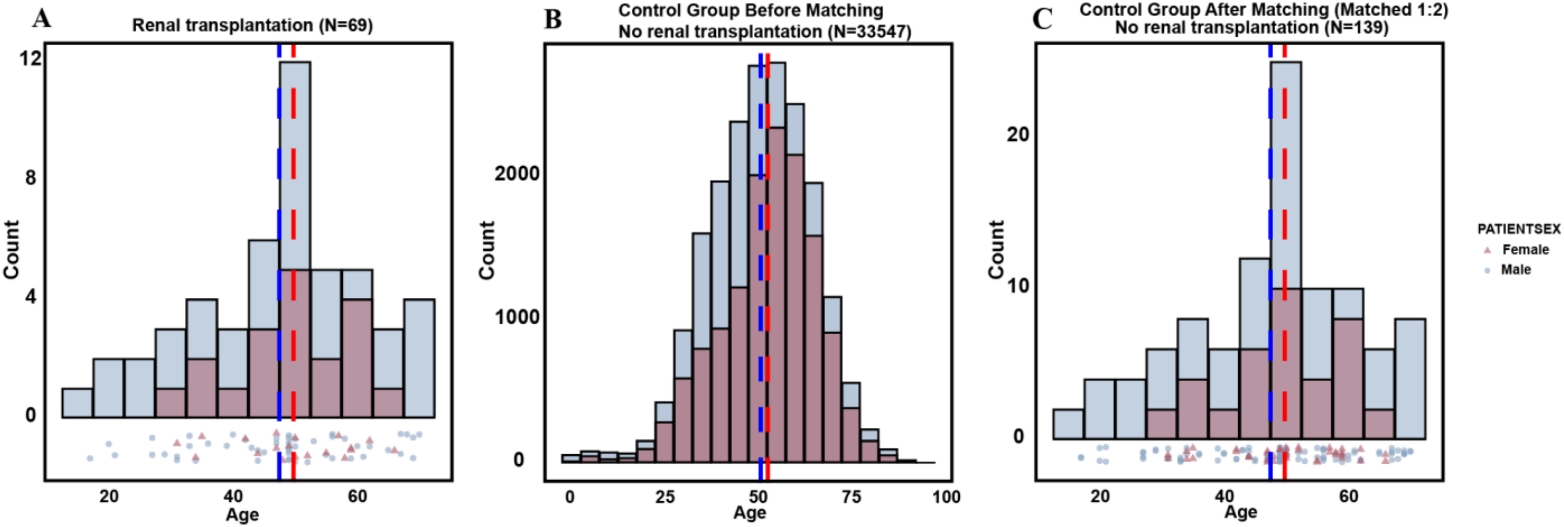
Comparison of age and sex distribution between kidney transplant and control groups before and after matching. **(A)** Kidney transplant group (N = 69). **(B)** Control group (non-transplant patients) before matching (N = 33,547). **(C)** Control group after 1:2 matching (N=139). The matched groups demonstrate improved comparability in demographic characteristics.

We further evaluated the age and sex distribution of the study cohorts (**Figure 2**). Among the 33,616 stone-forming patients from two hospitals, 69 underwent renal transplantation and subsequently developed nephrolithiasiswhile 33,547 stone-forming patients did not have a transplant history. Before matching, the control group demonstrated a substantially larger sample size with a broad age distribution centered around 50 years (mean age 51.59 ± 13.59 years), whereas the renal transplantation group showed a younger mean age of 48.12 ± 12.64 years (p=0.034) (**Figures 2A, B**). The renal transplantation group exhibited a male predominance (72.5% vs. 59.2%, p=0.034). After 1:2 propensity score matching, 139 non-transplant stone-forming patients were selected as controls, achieving excellent balance in age (48.13 ± 12.55 vs. 48.12 ± 12.64 years, p=0.994) and sex distribution (72.7% vs. 72.5% male, p=1.000), as demonstrated by the overlapping age distributions in the right panel of **Figure 2C**.

**Table 1** presents the pre-matching comparison, revealing significant differences in the distribution of stone composition between groups (p<0.001). The renal transplantation group showed a markedly higher proportion of carbonate apatite stones (37.7% vs. 16.4%) and calcium phosphate stones (8.7% vs. 2.7%), while calcium oxalate monohydrate stones were less prevalent (39.1% vs. 63.7%). Notably, ammonium urate and cystine stones were absent in the transplant cohort. Regarding comorbidities and urological conditions, the transplant group demonstrated significantly higher rates of urinary tract stricture (21.7% vs. 7.2%, p<0.001), renal insufficiency (15.9% vs. 8.4%, p=0.042), renal atrophy (14.5% vs. 5.5%, p=0.003), and hypertension (46.4% vs. 20.2%, p<0.001).

**Table 1:** Demographic characteristics, comorbidities, and stone compositions by renal transplantation status.

Group	Level	No renal transplantation (N = 33547)	Renal transplantation (N = 69)	*P*
type (%)	AU	85 (0.3)	0 (0.0)	<0.001
	CA	5486 (16.4)	26 (37.7)	
	CaOx	21369 (63.7)	27 (39.1)	
	CaP	920 (2.7)	6 (8.7)	
	Cystine Stone	213 (0.6)	0 (0.0)	
	MAP	1119 (3.3)	4 (5.8)	
	Others	27 (0.1)	0 (0.0)	
	Uric Acid Stone	4328 (12.9)	6 (8.7)	
Season (%)	Fall	8773 (26.2)	19 (27.5)	0.702
	Spring	8294 (24.7)	13 (18.8)	
	Summer	10257 (30.6)	24 (34.8)	
	Winter	6223 (18.6)	13 (18.8)	
PATIENTSEX (%)	Female	13687 (40.8)	19 (27.5)	0.034
	Male	19860 (59.2)	50 (72.5)	
PATIENTAGE (mean (SD))		51.59 (13.59)	48.12 (12.64)	0.034
StoneLoc (%)	Other Stones	17 (0.1)	0 (0.0)	0.055
	Kidney Stones	24376 (72.7)	61 (88.4)	
	Ureteral Stones	7980 (23.8)	6 (8.7)	
	Bladder Stones	1044 (3.1)	2 (2.9)	
	Posterior urethral stones	130 (0.4)	0 (0.0)	
UTI (%)	0	15790 (47.1)	37 (53.6)	0.333
	1	17757 (52.9)	32 (46.4)	
UTHE (%)	0	12438 (37.1)	33 (47.8)	0.085
	1	21109 (62.9)	36 (52.2)	
UTS (%)	0	31124 (92.8)	54 (78.3)	<0.001
	1	2423 (7.2)	15 (21.7)	
RI (%)	0	30728 (91.6)	58 (84.1)	0.042
	1	2819 (8.4)	11 (15.9)	
RF (%)	0	33264 (99.2)	68 (98.6)	1
	1	283 (0.8)	1 (1.4)	
RA (%)	0	31686 (94.5)	59 (85.5)	0.003
	1	1861 (5.5)	10 (14.5)	
UTM (%)	0	32846 (97.9)	69 (100.0)	0.428
	1	701 (2.1)	0 (0.0)	
HUA (%)	0	32454 (96.7)	68 (98.6)	0.613
	1	1093 (3.3)	1 (1.4)	
Gout (%)	0	33233 (99.1)	68 (98.6)	1
	1	314 (0.9)	1 (1.4)	
RC (%)	0	30748 (91.7)	67 (97.1)	0.157
	1	2799 (8.3)	2 (2.9)	
T/N (%)	0	32160 (95.9)	67 (97.1)	0.832
	1	1387 (4.1)	2 (2.9)	
NB (%)	0	33535 (100.0)	69 (100.0)	1
	1	12 (0.0)	0 (0.0)	
BPH (%)	0	31703 (94.5)	67 (97.1)	0.495
	1	1844 (5.5)	2 (2.9)	
NP (%)	0	33368 (99.5)	68 (98.6)	0.829
	1	179 (0.5)	1 (1.4)	
PUD (%)	0	33482 (99.8)	69 (100.0)	1
	1	65 (0.2)	0 (0.0)	
CTD (%)	0	33469 (99.8)	68 (98.6)	0.4
	1	78 (0.2)	1 (1.4)	
FLD (%)	0	33006 (98.4)	69 (100.0)	0.559
	1	541 (1.6)	0 (0.0)	
HT (%)	0	33398 (99.6)	69 (100.0)	1
	1	149 (0.4)	0 (0.0)	
HPT (%)	0	33506 (99.9)	69 (100.0)	1
	1	41 (0.1)	0 (0.0)	
HT- (%)	0	33477 (99.8)	69 (100.0)	1
	1	70 (0.2)	0 (0.0)	
A (%)	0	32967 (98.3)	68 (98.6)	1
	1	580 (1.7)	1 (1.4)	
DM (%)	0	30340 (90.4)	59 (85.5)	0.235
	1	3207 (9.6)	10 (14.5)	
HTN (%)	0	26759 (79.8)	37 (53.6)	<0.001
	1	6788 (20.2)	32 (46.4)	
HLP (%)	0	33011 (98.4)	69 (100.0)	0.564
	1	536 (1.6)	0 (0.0)	
COPD (%)	0	33415 (99.6)	69 (100.0)	1
	1	132 (0.4)	0 (0.0)	
CVD (%)	0	33183 (98.9)	69 (100.0)	0.774
	1	364 (1.1)	0 (0.0)	
CHD (%)	0	32826 (97.9)	67 (97.1)	0.989
	1	721 (2.1)	2 (2.9)	
HD (%)	0	32672 (97.4)	67 (97.1)	1
	1	875 (2.6)	2 (2.9)	
AD (%)	0	33528 (99.9)	69 (100.0)	1
	1	19 (0.1)	0 (0.0)	
Sepsis (%)	0	33439 (99.7)	69 (100.0)	1
	1	108 (0.3)	0 (0.0)	
SGC (%)	0	31596 (94.2)	69 (100.0)	0.071
	1	1951 (5.8)	0 (0.0)	
MS (%)	No	17855 (53.2)	29 (42.0)	0.082
	Yes	15692 (46.8)	40 (58.0)	

SL, Stone location; UTI, urinary tract infection; UTHE, urinary tract hydronephrosis and effusion; UTS, urinary tract stricture; RI, renal insufficiency; RF, renal failure; RA, renal atrophy; UTM, urinary tract malformation; HUA, hyperuricemia; RC, renal cyst; RT, renal transplantation; T/N, tumor/neoplasm; NB, neurogenic bladder; BPH, benign prostatic hyperplasia; NP, nephropathy; PUD, peptic ulcer disease; CTD, connective tissue disease; FLD, fatty liver disease; HT, hyperthyroidism; HPT, hyperparathyroidism; HT-, hypothyroidism; A, anemia; DM, diabetes mellitus; HTN, hypertension; HLP, hyperlipidemia; COPD, chronic obstructive pulmonary disease; CVD, cerebrovascular disease; CHD, coronary heart disease; HD, heart disease; AD, anxiety disorder; SGC, secondary growth of calculi; StC, stone composition; MS, mixed stones.

After propensity score matching (**Table 2**), the stone composition differences persisted and became even more pronounced (p=0.001), with the transplant group maintaining significantly higher proportions of carbonate apatite (37.7% vs. 15.8%) and calcium phosphate stones (8.7% vs. 2.9%), and lower calcium oxalate monohydrate prevalence (39.1% vs. 66.9%). The matched cohorts showed improved balance in most baseline characteristics, though significant differences remained in several variables. The transplant group continued to exhibit higher prevalence of urinary tract stricture (21.7% vs. 9.4%, p=0.025), renal atrophy (14.5% vs. 3.6%, p=0.01), diabetes mellitus (14.5% vs. 5.0%, p=0.038), and hypertension (46.4% vs. 15.8%, p<0.001). Additionally, stone location distribution differed significantly (p=0.011), with the transplant group showing a higher proportion of single-site stones (88.4% vs. 69.8%). Interestingly, the control group had a higher prevalence of staghorn calculi (8.6% vs. 0%, p=0.028), while the transplant group demonstrated a significantly higher proportion of mixed stones containing two or more different stone compositions (39.6% vs. 58.0%, p=0.018).

**Table 2:** Demographic characteristics, comorbidities, and stone compositions after propensity score matching (1:2).

Group	Level	No renal transplantation (N = 139)	Renal transplantation (N = 69)	*P*
type (%)	CA	22 (15.8)	26 (37.7)	0.001
	CaOx	93 (66.9)	27 (39.1)	
	CaP	4 (2.9)	6 (8.7)	
	Cystine Stone	1 (0.7)	0 (0.0)	
	MAP	3 (2.2)	4 (5.8)	
	Uric Acid Stone	16 (11.5)	6 (8.7)	
Season (%)	Fall	35 (25.2)	19 (27.5)	0.879
	Spring	33 (23.7)	13 (18.8)	
	Summer	47 (33.8)	24 (34.8)	
	Winter	24 (17.3)	13 (18.8)	
PATIENTSEX (%)	Female	38 (27.3)	19 (27.5)	1
	Male	101 (72.7)	50 (72.5)	
PATIENTAGE (mean (SD))		48.13 (12.55)	48.12 (12.64)	0.994
StoneLoc (%)	Kidney Stones	97 (69.8)	61 (88.4)	0.011
	Ureteral Stones	36 (25.9)	6 (8.7)	
	Bladder Stones	6 (4.3)	2 (2.9)	
UTI (%)	0	66 (47.5)	37 (53.6)	0.492
	1	73 (52.5)	32 (46.4)	
UTHE (%)	0	57 (41.0)	33 (47.8)	0.432
	1	82 (59.0)	36 (52.2)	
UTS (%)	0	126 (90.6)	54 (78.3)	0.025
	1	13 (9.4)	15 (21.7)	
RI (%)	0	129 (92.8)	58 (84.1)	0.084
	1	10 (7.2)	11 (15.9)	
RF (%)	0	137 (98.6)	68 (98.6)	1
	1	2 (1.4)	1 (1.4)	
RA (%)	0	134 (96.4)	59 (85.5)	0.01
	1	5 (3.6)	10 (14.5)	
UTM (%)	0	137 (98.6)	69 (100.0)	0.805
	1	2 (1.4)	0 (0.0)	
HUA (%)	0	137 (98.6)	68 (98.6)	1
	1	2 (1.4)	1 (1.4)	
Gout (%)	0	139 (100.0)	68 (98.6)	0.72
	1	0 (0.0)	1 (1.4)	
RC (%)	0	127 (91.4)	67 (97.1)	0.208
	1	12 (8.6)	2 (2.9)	
T/N (%)	0	135 (97.1)	67 (97.1)	1
	1	4 (2.9)	2 (2.9)	
NB (%)	0	139 (100.0)	69 (100.0)	NA
BPH (%)	0	129 (92.8)	67 (97.1)	0.35
	1	10 (7.2)	2 (2.9)	
NP (%)	0	139 (100.0)	68 (98.6)	0.72
	1	0 (0.0)	1 (1.4)	
PUD (%)	0	139 (100.0)	69 (100.0)	NA
CTD (%)	0	139 (100.0)	68 (98.6)	0.72
	1	0 (0.0)	1 (1.4)	
FLD (%)	0	137 (98.6)	69 (100.0)	0.805
	1	2 (1.4)	0 (0.0)	
HT (%)	0	139 (100.0)	69 (100.0)	NA
HPT (%)	0	139 (100.0)	69 (100.0)	NA
HT- (%)	0	138 (99.3)	69 (100.0)	1
	1	1 (0.7)	0 (0.0)	
A (%)	0	138 (99.3)	68 (98.6)	1
	1	1 (0.7)	1 (1.4)	
DM (%)	0	132 (95.0)	59 (85.5)	0.038
	1	7 (5.0)	10 (14.5)	
HTN (%)	0	117 (84.2)	37 (53.6)	<0.001
	1	22 (15.8)	32 (46.4)	
HLP (%)	0	137 (98.6)	69 (100.0)	0.805
	1	2 (1.4)	0 (0.0)	
COPD (%)	0	138 (99.3)	69 (100.0)	1
	1	1 (0.7)	0 (0.0)	
CVD (%)	0	138 (99.3)	69 (100.0)	1
	1	1 (0.7)	0 (0.0)	
CHD (%)	0	136 (97.8)	67 (97.1)	1
	1	3 (2.2)	2 (2.9)	
HD (%)	0	136 (97.8)	67 (97.1)	1
	1	3 (2.2)	2 (2.9)	
AD (%)	0	139 (100.0)	69 (100.0)	NA
Sepsis (%)	0	138 (99.3)	69 (100.0)	1
	1	1 (0.7)	0 (0.0)	
SGC (%)	0	127 (91.4)	69 (100.0)	0.028
	1	12 (8.6)	0 (0.0)	
MS (%)	No	84 (60.4)	29 (42.0)	0.018
	Yes	55 (39.6)	40 (58.0)	

### Temporal and demographic patterns of urinary stone formation in patients

**Figure 3:**
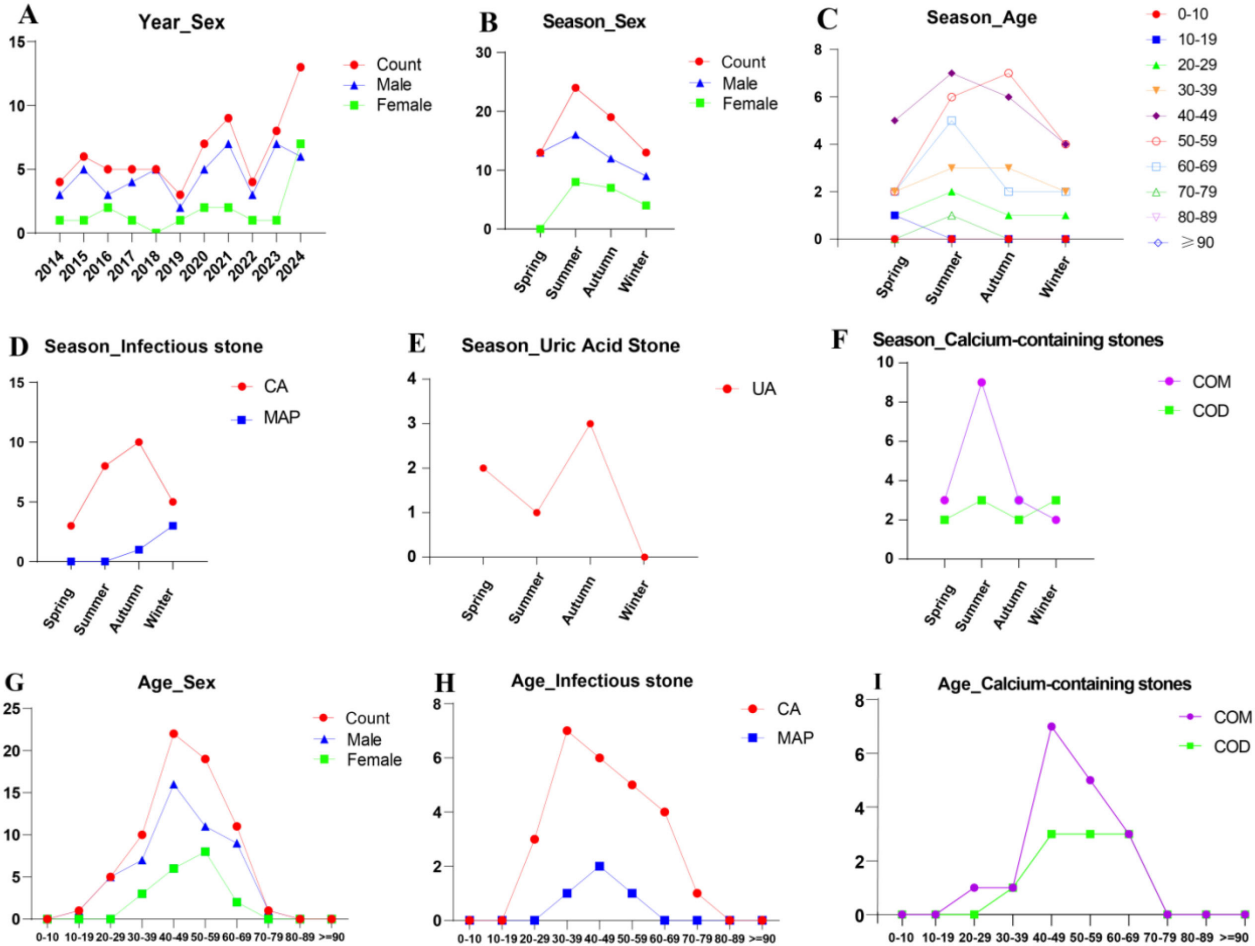
Comprehensive demographic and compositional profile of the 69 kidney transplant recipients with urinary stones. **(A)** Annual case distribution by sex. **(B)** Seasonal case distribution by sex. **(C)** Seasonal case distribution across age groups (0-10, 10-19, 20-29, 30-39, 40-49, 50-59, 60-69, 70-79, 80-89, ≥90 years). **(D)** Seasonal distribution of infectious stone components: carbonate apatite (CA) and magnesium ammonium phosphate (MAP). **(E)** Seasonal distribution of uric acid (UA) stones. **(F)** Seasonal distribution of calcium-containing stones: calcium oxalate monohydrate (COM) and calcium oxalate dihydrate (COD). **(G)** Overall age distribution by sex. **(H)** Age-specific distribution of infectious stone components (CA, MAP). **(I)** Age-specific distribution of calcium-containing stones (COM, COD).

Analysis of the 69 kidney transplant recipients with urinary stones revealed distinctive patterns. The cohort showed consistent male predominance (72.5%) across the study period, with most cases occurring in patients aged 40-49 (n=22) (**Figure 3G**). The cohort showed consistent male predominance throughout the study period, with case numbers increasing notably after 2019 (**Figure 3A**). Stone presentation exhibited seasonal variation, with higher frequency in summer (n=24) and autumn (n=19) compared to spring (n=14) and winter (n=14) (**Figure 3B**). This pattern was particularly evident in patients aged 40–69 years (**Figure 3C**). Compositionally, infection-related stones were prominent, with carbonate apatite (CA) representing the most frequent infectious component across seasons (**Figure 3D**). CA stones were most prevalent in younger transplant recipients, peaking in the 30–39 year age group (n=7) (**Figure 3H**). Calcium-containing stones, particularly calcium oxalate monohydrate (COM), showed higher counts in summer (n=9) and were most common in patients aged 40-49 (n=7) and 50-59 (n=5) years (**Figures 3F, I**). Uric acid stones demonstrated seasonal variation with highest frequency in autumn (n=3) (**Figure 3E**).

### Univariate associations with stone composition

**Figure 4:**
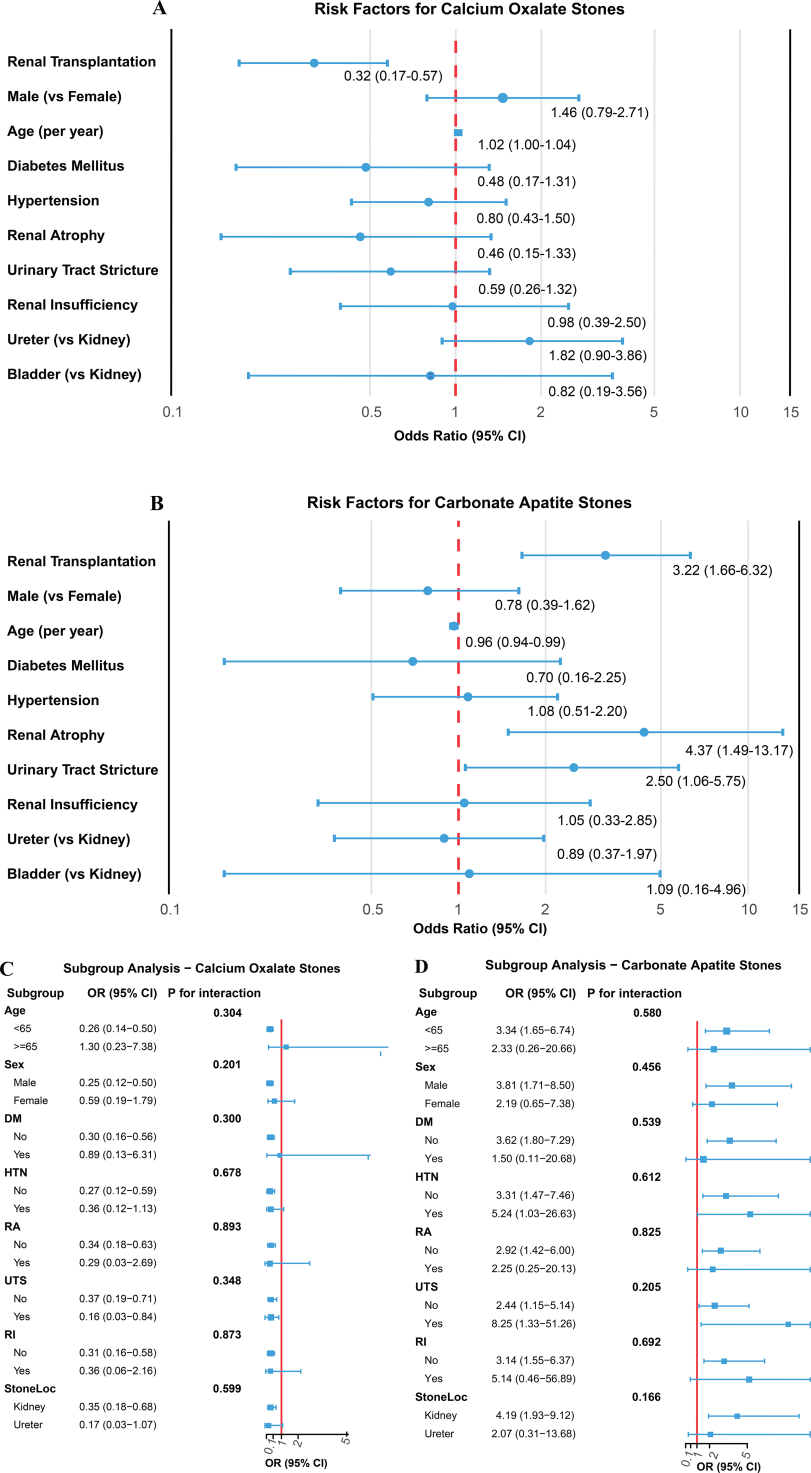
Risk factors and subgroup analyses for calcium oxalate and carbonate apatite stones. **(A)** Risk Factors for Calcium Oxalate Stones: Forest plot displaying odds ratios for clinical factors associated with calcium oxalate stone formation. **(B)** Risk Factors for Carbonate Apatite Stones: Forest plot displaying odds ratios for clinical factors associated with carbonate apatite stone formation. **(C)** Subgroup Analysis for Calcium Oxalate Stones: Assessment of effect modification by patient demographics and comorbidities. **(D)** Subgroup Analysis for Carbonate Apatite Stones: Assessment of effect modification by patient demographics and comorbidities.

Univariate logistic regression analysis revealed that renal transplantation was significantly associated with specific stone types (**Figure 4**). Specifically, renal transplantation demonstrated a strong inverse association with calcium oxalate stone formation (OR 0.32, 95% CI: 0.17-0.57, p<0.001), indicating that among stone-forming patients, transplant recipients had approximately 68% lower odds of having a stone dominated by calcium oxalate compared to matched non-transplant stone formers. Conversely, renal transplantation was significantly associated with a higher odds of of carbonate apatite stone formation (OR 3.22, 95% CI: 1.66-6.32, p<0.001), representing more than a three-fold higher odds of having a carbonate apatite-dominant stone in the transplant group. These findings remained consistent with the compositional differences observed in the baseline comparison, reinforcing the distinct stone composition profiles in renal transplant stone formers.

For carbonate apatite stones, additional clinical factors associated with carbonate apatite stones emerged beyond transplantation status. Renal atrophy showed the strongest association (OR 4.37, 95% CI: 1.49-13.17, p=0.007), followed by urinary tract stricture (OR 2.50, 95% CI: 1.06-5.75, p=0.032), both suggesting that structural and anatomical abnormalities of the urinary system contribute to carbonate apatite stone formation in patients who develop stones. Interestingly, younger age was also inversely associated with carbonate apatite stones (OR 0.96 per year, 95% CI: 0.94-0.99, p=0.006). Other variables including sex, diabetes mellitus, hypertension, renal insufficiency, and stone location showed no significant associations with either stone type in the univariate analysis.

### Subgroup analysis of renal transplantation effect on calcium oxalate stone formation

To explore whether the inverse association between renal transplantation and calcium oxalate stones varied across different patient subgroups, we conducted comprehensive subgroup analyses stratified by demographic characteristics, comorbidities, and urological conditions (**Figure 4**). This inverse association of renal transplantation with calcium oxalate stones was consistently observed across most subgroups, though the magnitude of the association varied. In patients younger than 65 years, renal transplantation demonstrated a particularly strong inverse association (OR 0.26, 95% CI: 0.14-0.50), whereas this association was attenuated and lost statistical significance in older patients (≥65 years: OR 1.30, 95% CI: 0.23-7.38), though the interaction was not statistically significant (p=0.304). Similarly, the inverse association appeared more pronounced in male patients (OR 0.25, 95% CI: 0.12-0.50) compared to female patients (OR 0.59, 95% CI: 0.19-1.79), but again without significant interaction (p=0.201).

The inverse association remained robust across subgroups defined by comorbidities and urological conditions, with no significant effect modification detected. Among patients without diabetes mellitus (OR 0.30, 95% CI: 0.16-0.56), hypertension (OR 0.27, 95% CI: 0.12-0.59), renal atrophy (OR 0.34, 95% CI: 0.18-0.63), urinary tract stricture (OR 0.37, 95% CI: 0.19-0.71), or renal insufficiency (OR 0.31, 95% CI: 0.16-0.58), renal transplantation consistently showed inverse associations with calcium oxalate stones. Notably, the inverse association was even more pronounced in patients with urinary tract stricture (OR 0.16, 95% CI: 0.03-0.84), though the interaction test was not significant (p=0.348). Stone location also did not significantly modify the transplantation association (p=0.599), with inverse associations observed in both kidney stones (OR 0.35, 95% CI: 0.18-0.68) and ureteral stones (OR 0.17, 95% CI: 0.03-1.07). The consistency of these findings across diverse patient subgroups strengthens the evidence that renal transplantation fundamentally alters the stone composition profile, specifically characterized by a lower representation of calcium oxalate stones, in patients who develop nephrolithiasis.

### Sensitivity analysis with progressive adjustment for confounders

**Figure 5:**
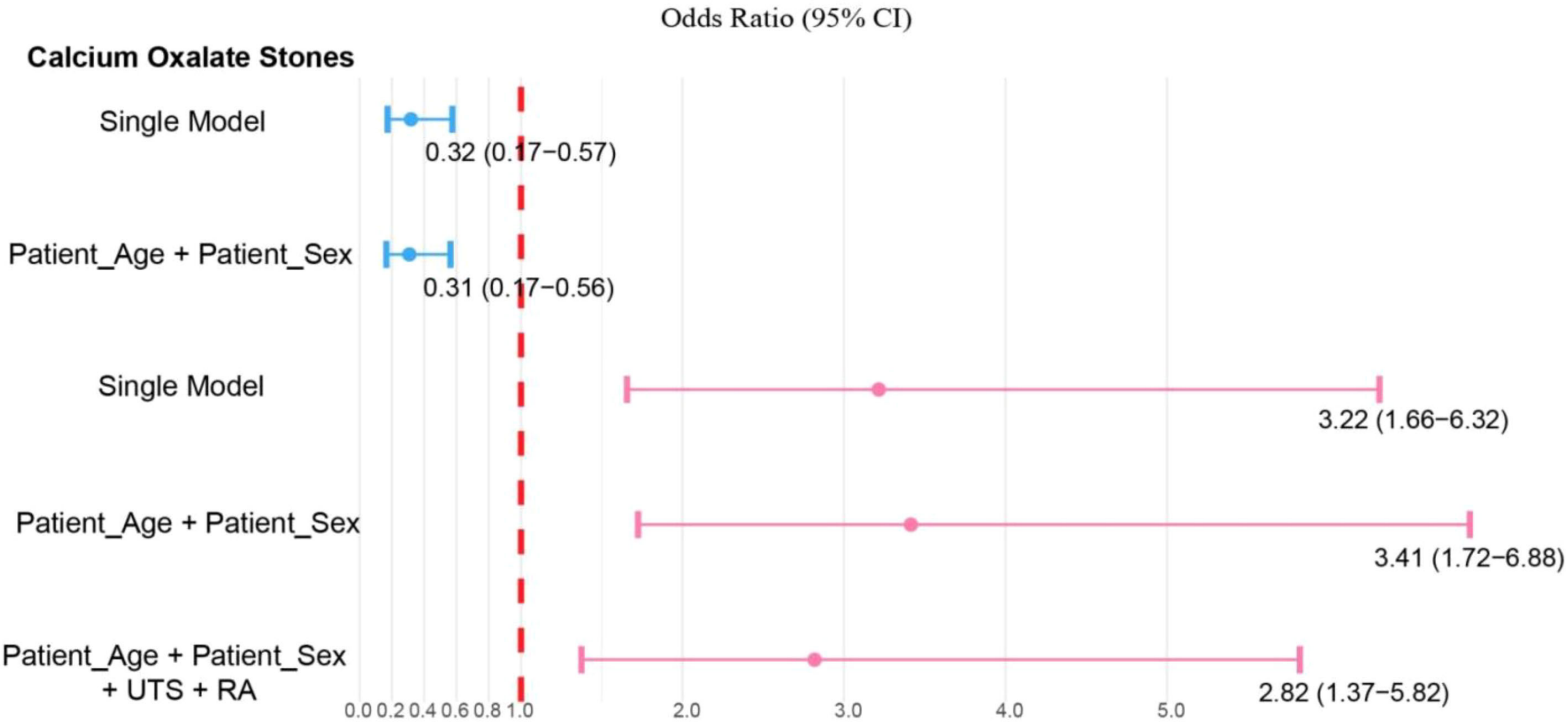
Multivariate logistic regression analysis for calcium oxalate stones and carbonate apatite stones. Results of multivariate models showing adjusted odds ratios (OR) with 95% confidence intervals for factors associated with Calcium Oxalate Stones and Carbonate Apatite Stones.

To assess the robustness of the observed associations and examine potential confounding effects, we performed sensitivity analyses using progressively adjusted logistic regression models (**Figure 5**). For the lower proportion of calcium oxalate stones among stone formers, the inverse association with renal transplantation remained consistent across different modeling strategies. In the unadjusted model, renal transplantation showed a strong inverse association with calcium oxalate stones (OR 0.32, 95% CI: 0.17-0.57), which persisted after adjusting for age and sex (OR 0.31, 95% CI: 0.17-0.56), demonstrating minimal confounding by these demographic factors. The consistency of effect estimates across models suggests that the inverse association between renal transplantation and calcium oxalate stone formation is robust and unlikely to be explained by age or sex differences between groups.

In contrast, the positive association between renal transplantation and carbonate apatite stones showed more notable changes with progressive adjustment. The unadjusted model revealed a strong association (OR 3.22, 95% CI: 1.66-6.32), which remained significant after adjusting for age and sex (OR 3.41, 95% CI: 1.72-6.88). When further adjusted for urinary tract stricture and renal atrophy—two factors independently associated with carbonate apatite formation—the association was attenuated but remained statistically significant (OR 2.82, 95% CI: 1.37-5.82). This attenuation suggests that structural and anatomical abnormalities of the urinary tract may partially mediate the relationship between renal transplantation and carbonate apatite stone formation. Nevertheless, the persistence of a significant association even after accounting for these factors indicates that renal transplantation is linked to additional effects on carbonate apatite stone occurrence beyond its association with urological complications.

## Discussion

This comprehensive analysis of 33,616 urinary stone cases represents one of the largest comparative studies examining stone composition patterns in renal transplant recipients versus matched controls. Our principal findings demonstrate that the stone composition profile differs fundamentally in transplant recipients, who exhibit a 3.2-fold higher odds of carbonate apatite stones (OR 3.22, 95% CI: 1.66-6.32, p<0.001) and 68% lower odds of calcium oxalate stones (OR 0.32, 95% CI: 0.17-0.57, p<0.001) compared to propensity score-matched non-transplant stone formers. These associations remained robust across multiple sensitivity analyses and diverse patient subgroups. Additionally, transplant recipients demonstrated significantly higher proportions of mixed stone composition (58.0% vs. 39.6%, p=0.018), urinary tract stricture (21.7% vs. 9.4%, p=0.025), and renal atrophy (14.5% vs. 3.6%, p=0.01). These findings challenge the conventional understanding that calcium oxalate stones dominate across all patient populations and underscore the unique metabolic and anatomical environment created by transplantation in this stone-forming cohort.

The strikingly higher proportion of carbonate apatite stones in our transplant cohort (37.7% vs. 15.8% in controls) aligns with emerging evidence that post-transplant metabolic bone disease and altered calcium-phosphate homeostasis create a permissive environment for phosphate-based stone formation. Recent studies have identified that chronic immunosuppressive therapy, particularly calcineurin inhibitors and corticosteroids, disrupts normal bone metabolism and increases urinary phosphate excretion (17, 18). Ganesan et al. (2023) reported that kidney stone events affect 2.6% of transplant recipients within 5 years post-transplantation, with infection-related stones being disproportionately represented (5). The immunosuppressive regimens used in transplantation—including corticosteroids, calcineurin inhibitors, and antiproliferative agents—create a complex metabolic milieu that extends beyond their intended immunomodulatory effects. For instance, glucocorticoids such as methylprednisolone, commonly used in both solid organ and hematopoietic stem cell transplantation protocols, exert profound effects on calcium-phosphate metabolism through multiple mechanisms including increased renal calcium excretion, decreased intestinal calcium absorption, and direct effects on osteoblast and osteoclast function (19). Our findings extend previous observations by quantifying the magnitude of this association and identifying structural urological complications—particularly urinary tract stricture (OR 2.50, p=0.032) and renal atrophy (OR 4.37, p=0.007)—as independent factors associated with carbonate apatite stones. The association between urinary tract stricture and carbonate apatite stones likely reflects urinary stasis and altered urodynamics in transplanted kidneys, which promote crystal nucleation and growth in alkaline urine environments (20). Furthermore, post-transplant hyperparathyroidism, which can persist despite restored renal function, contributes to hypercalciuria and hyperphosphaturia, providing the biochemical substrate for carbonate apatite crystallization (21, 22). A 2024 systematic review by Piana et al. emphasized that urological complications and metabolic derangements create a “perfect storm” for atypical stone formation in transplant recipients (9). The role of chronic low-grade inflammation in the transplanted kidney, mediated by subclinical rejection episodes and ischemia-reperfusion injury, may further contribute to localized changes in urinary pH and crystallization inhibitors that favor phosphate stone formation (23, 24).

The striking inverse association between renal transplantation and calcium oxalate stones (OR 0.32) represents a novel finding that contradicts the historical assumption that calcium oxalate stones remain predominant across all patient populations. This observed compositional shift may be mechanistically explained by several post-transplant metabolic alterations. First, the restoration of renal function following successful transplantation normalizes many uremic metabolic derangements that promote calcium oxalate crystallization, including improved citrate excretion—a natural stone inhibitor (25, 26). The improvement in glomerular filtration following transplantation fundamentally alters the urinary milieu, potentially reversing the hypocitraturia and metabolic acidosis that characterize chronic kidney disease (27). Second, immunosuppressive medications, particularly calcineurin inhibitors, have been shown to alter tubular handling of calcium and oxalate, potentially reducing supersaturation of calcium oxalate in urine (28, 29). Recent metabolomic profiling studies have demonstrated that transplant recipients exhibit distinct urinary metabolite signatures compared to non-transplant stone formers, with reduced urinary oxalate concentrations despite comparable dietary oxalate intake (30). Additionally, post-transplant dietary modifications, including reduced sodium and protein intake recommended for graft preservation, may inadvertently reduce calcium oxalate supersaturation (31). The widespread use of mycophenolate mofetil in modern immunosuppressive regimens may also play a role in reducing calcium oxalate stone propensity through its effects on purine metabolism and subsequent alterations in urinary chemistry (32, 33). Our subgroup analyses revealed that this inverse association was particularly pronounced in younger patients (<65 years: OR 0.26) and males (OR 0.25), suggesting that metabolic factors influenced by age and sex hormones may effect of transplantation on stone composition The more pronounced association in younger patients may reflect better graft function, more aggressive immunosuppression protocols, and greater adherence to dietary recommendations in this demographic (34, 35). Importantly, the consistency of this association across diverse subgroups—including those with diabetes, hypertension, and various urological conditions—strengthens the evidence that transplantation-related metabolic changes, rather than selection bias or confounding, underlie the observed shift in stone composition profile. The proportion of patients with diabetes mellitus was notably higher in our transplant cohort (14.5% vs. 5.0%, p=0.038), and emerging evidence suggests complex interactions between diabetic kidney disease and stone disease, with diabetic metabolic derangements potentially influencing both stone composition and recurrence patterns (36).

The significantly higher proportion of mixed stones in transplant recipients (58.0% vs. 39.6%, p=0.018) represents an important clinical challenge that reflects the complex pathophysiology in this population. A detailed analysis of mixed stones revealed that, among carbonate apatite (CA)-dominant stones, calcium oxalate monohydrate (COM) was the most frequent secondary component. This was observed in 34.6% of CA-dominant stones in transplant recipients and 40.9% in matched controls. Mixed stones, containing two or more distinct compositional components (e.g., calcium oxalate combined with carbonate apatite), indicate that multiple lithogenic mechanisms are simultaneously active (11, 37). This compositional heterogeneity likely arises from immunosuppression-induced metabolic alterations, fluctuating urine pH, anatomical abnormalities, and polypharmacy effects that create variable conditions for different crystal types (2, 38). The layered architecture of mixed stones reflects sequential crystallization processes driven by changing metabolic conditions over time. In line with this, we found that calcium oxalate monohydrate was the predominant secondary component in carbonate apatite-dominant stones (34.6% in transplant recipients and 40.9% in controls), underscoring the common sequence of crystallization where an alkaline, infection-promoting environment may subsequently favor calcium oxalate precipitation. Our observation that transplant recipients had no staghorn calculi (0% vs. 8.6%, p=0.028) but higher proportions of single-site stones (88.4% vs. 69.8%, p=0.011) suggests distinct stone growth dynamics, The absence of staghorn calculi may be attributed to a combination of factors: the close nephrological and radiological surveillance that transplant recipients undergo, leading to earlier detection and intervention before stones can grow extensively, along with effective infection control or altered bacterial colonization patterns in the context of prophylactic antimicrobial strategies and immunosuppression (39, 40). From a management perspective, mixed stones pose therapeutic challenges as different components require contradictory treatments—carbonate apatite resists medical dissolution while calcium oxalate management may require opposing pH modifications (41, 42). Moreover, surgical intervention in transplanted kidneys presents unique risks including altered anatomy, immunosuppression-related bleeding, and graft injury concerns (6, 43). Recent advances in miniaturized ureteroscopy have improved outcomes, though complication rates remain elevated compared to native kidneys (44).

Our study’s principal strengths include the large sample size spanning a decade, rigorous propensity score matching to minimize selection bias, and validated stone composition analysis using Fourier Transform-Infrared Spectrometry. However, limitations include the retrospective design precluding definitive causal conclusions, limited generalizability from our Southern China population, relatively small transplant recipient cohort (n=69), and lack of detailed immunosuppressive regimen data and longitudinal urinary metabolic parameters. Furthermore, our dataset did not capture information on the use of indwelling urinary devices (e.g., double-J stents or nephrostomy tubes), which are established risk factors for infection and stone formation. Their more frequent use in transplant recipients could be an additional, unmeasured contributor to the higher proportion of infection stones observed in this group. Most importantly, as our study population comprised exclusively patients who had already formed stones, our findings characterize the composition of stones in transplant recipients but cannot address whether transplantation alters the overall incidence of nephrolithiasis. In addition, our classification of all carbonate apatite stones as infection stones, while following EAU guidelines (13), may overestimate infectious etiology in individual cases where non-infectious alkaline urine (e.g., renal tubular acidosis) is present. Future research should first address whether renal transplantation alters the incidence of nephrolithiasis. This requires prospective cohort studies comparing transplant recipients with appropriate control populations (e.g., patients with advanced chronic kidney disease and healthy individuals) using standardized surveillance for symptomatic and asymptomatic stone events. Once the incidence question is resolved, prospective designs with serial metabolic evaluations, including 24-hour urine chemistry analyses, to elucidate temporal evolution of stone stone-forming propensity post-transplantation (45). Future studies should also systematically document the frequency and modality of post-transplant radiological surveillance to quantify the contribution of early stone detection to observed stone burden and composition patterns. In addition, prospective collection of indwelling urinary device history—including stent type, indwelling duration, and concurrent antimicrobial prophylaxis—is essential to disentangle the effects of device-related infection from host metabolic factors. Investigation of the gut-kidney axis and microbiome alterations could reveal novel pathways linking immunosuppression to altered oxalate metabolism and stone formation (46, 47). These findings advocate for transplant-specific stone surveillance protocols with baseline metabolic screening, tailored dietary counseling, and consideration of prophylactic measures such as potassium citrate supplementation for high-risk recipients (48).

## Data Availability

The original contributions presented in the study are included in the article/**Supplementary Material**, further inquiries can be directed to the corresponding author/s.
