# Correction: Analysis of 33,616 urinary stone cases: novel findings on renal transplantation impact, comorbidity profiles, and composition patterns

**DOI:** 10.3389/fimmu.2026.1847065

**Published:** 2026-04-20

**Authors:** Lian Peng, Xingyu Zhou, Jialin Liu, Jierong Chen, Jiale Zhang, Feiyang Tan, Baolin Li, Ying Liang, Qianjun Li, Zhenglin Chang, Lin Yu, Ming Zhao

**Affiliations:** 1 Department of Urology, Zhujiang Hospital of Southern Medical University, Guangzhou, Guangdong, China; 2 Department of Urology, Yuebei People’s Hospital Affiliated to Shantou University Medical College, Shaoguan, China; 3 Department of Urology and Guangdong Key Laboratory of Urology, The First Affiliated Hospital of Guangzhou Medical University, Guangzhou, China; 4 Department of Clinical Laboratory, National Center for Respiratory Medicine, National Clinical Research Center for Respiratory Disease, State Key Laboratory of Respiratory Disease, Guangzhou Institute of Respiratory Health, The First Affiliated Hospital of Guangzhou Medical University, Guangzhou, Guangdong, China

**Keywords:** calcium oxalate, carbonate apatite, propensity score matching, renal transplantation, stone disease, urinary stone composition

Affiliation “Department of Urology, Zhujiang Hospital of Southern Medical University, Guangzhou, China” was omitted for authors Lian peng and Ming Zhao. This affiliation has now been added for authors Lian peng and Ming Zhao. AND Author Ming Zhao was erroneously assigned to the second affiliation (Department of Urology, Yuebei People's Hospital Affiliated to Shantou University Medical College, Shaoguan 512025, China). The correct affiliation is only the first one (Department of Urology, Zhujiang Hospital of Southern Medical University, Guangzhou 510280, China).

The original version of this article has been updated.

The institutional description in the **Materials and methods** section of our manuscript. While the data source presented in **Figure 1** is correct, we have identified an error in the text regarding the collaborating institution. The sentence currently reads: “This retrospective observational study analyzed urinary stone composition data from 33,616 samples collected at the First Affiliated Hospital of Guangzhou Medical University and Yuebei People’s Hospital of Shaoguan in Southern China between April 2014 and December 2024.” The correct institution should be Zhujiang Hospital of Southern Medical University, not Yuebei People’s Hospital of Shaoguan. All other details, including the data source and **Figure 1**, remain accurate.

A correction has been made to the section **Methods**, *Study design and sample collection,* Paragraph 1

“This retrospective observational study analyzed urinary stone composition data from 33,616 samples collected at the First Affiliated Hospital of Guangzhou Medical University and Zhujiang Hospital of Southern Medical University between April 2014 and December 2024.”

The original version of this article has been updated.

